# Metal–Phenolic Networks for Sensing Applications

**DOI:** 10.3390/bios15090600

**Published:** 2025-09-11

**Authors:** Ning Xia, Sirui Liang, Dehua Deng, Yong Chang, Xinyao Yi

**Affiliations:** 1 Henan Province Key Laboratory of New Opto-Electronic Functional Materials, College of Chemistry and Chemical Engineering, Anyang Normal University, Anyang 455000, China; lsr052178@163.com (S.L.); ddh@aynu.edu.cn (D.D.); yongchang_swudc@163.com (Y.C.); 2 College of Chemistry and Chemical Engineering, Central South University, Changsha 410083, China

**Keywords:** metal-phenolic networks, polyphenols, self-assembly, sensing, biosensors

## Abstract

The preparation of new inorganic–organic hybrid materials is beneficial for the development of powerful sensing methods and technologies. Polyphenols, a type of organic molecule containing phenolic hydroxyl groups, are widely present in natural plants and have beneficial effects on human health. Metal ions are ubiquitous in nature and play an important role in the development of inorganic–organic hybrid materials. Metal–phenolic networks (MPNs) are formed by the self-assembly of metal ions and polyphenols through dynamic coordination bonds. Due to their mild synthesis conditions, facilely engineered functionalities, and multiple modification strategies, MPNs have become potential platforms for sensing applications. Timely understanding of the function and application of MPNs in sensing fields will facilitate the development of novel chemical and biological sensors and devices. This article summarizes the typical preparation methods and excellent advantages of MPNs and focuses on their latest achievements in sensing applications. We highlight representative MPN-based sensing examples, including the direct detection of small molecules and biological species, immunoassays, bioimaging, and wearable devices. Finally, the prospects and future directions of MPNs in sensing fields are addressed.

## 1. Introduction

The main task of bioanalytical chemistry is to develop sensing methods for quantitatively monitoring the level of pollutants, biomarkers, drugs, and microenvironments [1]. A variety of detection techniques have been developed for sensitive and accurate quantitative detection, such as enzyme-linked immunosorbent assay, mass spectroscopy, and high-performance liquid chromatography. However, these methods always suffer from practical limitations of high costs, expensive instruments, and skillful operators, and their sensitivity cannot meet the demand of ultrasensitive, on-site, and real-time detection of low-abundance analytes [2]. The preparation of functional materials with tailored properties is vital for developing ultrasensitive and accurate sensing methods [3]. Until now, various materials have been exploited to improve detection sensitivity, including noble metal nanostructures, carbon-based nanomaterials, metal oxide/sulfide nanosheets, and inorganic–organic hybrid materials [4,5,6]. Among them, inorganic–organic hybrid materials formed by the assembly of organic and inorganic building elements have aroused extensive interests due to their excellent optical, chemical, electrochemical, and biological characteristics [7,8,9,10,11,12,13]. Metal–organic frameworks (MOFs), crystalline porous coordination polymers, and metal–phenolic networks (MPNs) are the three main types of inorganic–organic hybrid materials. Among them, MOFs have received considerable attention in various applications such as gas separation and storage, catalysis, biosensing, and biomedicine [14,15,16]. However, some MOFs have several inherent drawbacks in sensing applications, such as poor biocompatibility and harsh synthesis conditions (e.g., high temperatures and pressure, toxic organic reagent) [17].

**Figure 1:**
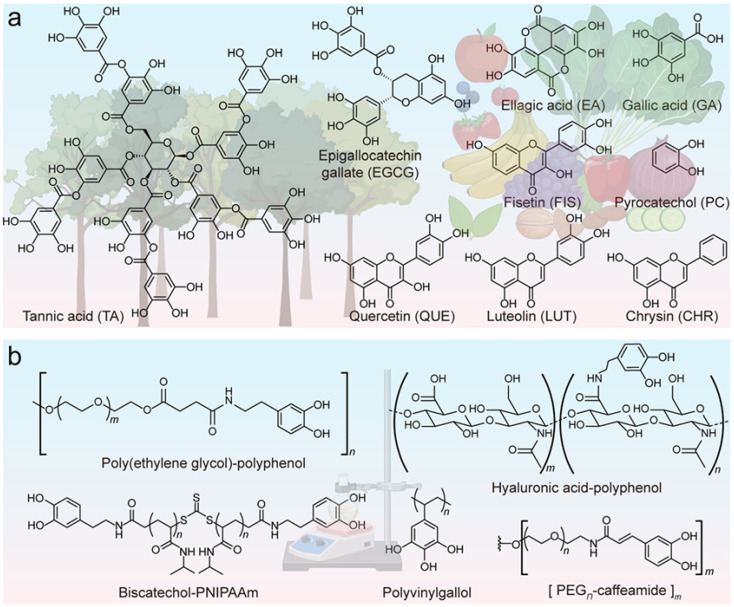
Representative examples of (**a**) natural and (**b**) synthetic phenolic ligands [24]. Reproduced from Ref. [24] with permission from the Royal Society of Chemistry.

MPNs are amorphous coordination networks formed by metal ions and polyphenols through dynamic coordination interactions [18]. They have attracted considerable attention in the fields of sensing, biomedicine, and interface engineering because of their remarkable advantages, such as catalytic activity, metal ion complexation, thermal stability, and optical properties [19,20,21]. As the molecular building blocks of MPNs, polyphenols are abundant, with approximately 8000 natural molecules and numerous synthetic ligands (Figure 1) [22,23,24]. Natural polyphenols can be mainly classified into four categories: lignans, stilbenes, phenolic acids, and flavonoids. Notably, tannic acid (TA), dopamine (DA), epigallocatechin gallate (EGCG), and gallic acid (GA) are the four popularly used polyphenol substances [25]. The catechol and gallol groups allow polyphenols to interact with various materials or substrates via covalent coupling, hydrogen bonding, π-π stacking, electrostatic, hydrophobic, and metal coordination interactions [26]. For example, inspired by the strong adhesion capability of mussel foot proteins, DA has been widely used to coat diverse inorganic and organic substrates by self-assembly into polydopamine [27,28]. The polymerization of DA can be achieved without the use of metal ions. The resulting polydopamine has been widely used in a variety of applications, such as chemistry, biomedicine, and material science. Since polydopamine does not belong to the family of MPNs, this review does not discuss its application, but interested researchers can refer to its relevant professional reviews [29,30]. In addition, to further tailor the properties and functions, specific molecules can be covalently conjugated with natural polyphenols such as polyethylene glycol and hyaluronic acid to form biomacromolecules. The polyphenol derivatives usually maintain their ability to coordinate with metal ions and intrinsic physicochemical properties.

Metal ions play an important role in the formation of MPNs, which typically include main group metal ions such as Al^3+^, transition metal ions (e.g., Fe^3+^, Cu^2+^, Co^2+^, and Ni^2+^), and lanthanide ions (e.g., Ce^3+^, Eu^3+^, and Ga^3+^). The type and valency and the molar ratio of metal ions to phenolic groups can regulate the stoichiometry and chemical properties of MPNs. More importantly, the selection of metal ions can modulate the function of as-synthesized MPNs [31,32,33]. For example, Cu^2+^ and Fe^3+^-based MPNs exhibit strong catalytic activity toward the Fenton reaction to produce reactive oxygen species (ROS), making them highly prevalent in the design of colorimetric biosensors [34]. Some transition metal ions (e.g., Cd^2+^, Pb^2+^, and Cu^2+^) in MPNs can be electrochemically detected for a signal readout with high sensitivity and simplicity. Europium (III) ions can endow the possibility of MPNs to fluorescence-guided multiplex phototherapy. The phenolic groups in polyphenols are pH-dependent polyvalent chelating sites for metal ions. Thus, the assembly and disassembly of MPNs can be modulated by the pH change in the surrounding environment, which is particularly popular in biomedical applications. Briefly, the increased alkalinity of the solution will facilitate the deprotonation of phenolic groups and the formation of coordination bonds. However, the phenolic groups are readily protonated under acidic conditions, resulting in the breaking of coordination bonds between phenolic groups and metal ions and, eventually, the dissociation of MPNs. Therefore, it is possible to synthesize different morphological materials with 2D or 3D geometries based on the molecular self-assembly of metal ions and polyphenols [24]. The abundant types of metal ions and polyphenols endow MPNs with distinctive properties and functions, making them promising alternative materials for sensing applications.

Up to now, many reviews have comprehensively summarized the advancements of MPNs in material engineering, interfacial modification, and biomedical research [28,33,35,36,37,38,39,40]. For example, Ejima et al. discussed the self-assembly process and mechanism, property, and application of MPN coatings on nanomaterials and substrate interfaces [20]. Lin et al. addressed the progress and application of MPNs in biomedicine [41]. Liu et al. reviewed the functions of MPNs and polyphenol derivatives in photo (electro) catalysis [42]. However, to the best of our knowledge, there are few reviews that systematically summarize the sensing applications of MPNs. In this review, we provide a short introduction to the commonly used metal ions and polyphenols for MPNs and then discuss the preparation methods (e.g., direct self-assembly, hard template-assisted self-assembly, emulsion-based interfacial self-assembly, and coating on substrate interfaces) and advantages of MPNs (e.g., mild synthesis conditions, facilely engineered functionalities, and multiple modification strategies). Thereafter, we mainly focus on the applications of MPNs in sensing fields, including the direct detection of small molecules and biological species, immunoassays, bioimaging, and wearable devices. Finally, we briefly summarize this work and look forward to the future challenges and directions related to the sensing applications of MPNs. This review is expected to provide fundamental and timely understanding in metal–phenolic systems and guide the design of novel inorganic–organic hybrid materials for sensing applications.

## 2. Synthesis and Advantages of MPNs

The synthesis of MPNs is usually simple, facile, and efficient. Under alkaline conditions, catechol and galloyl groups in polyphenols can undergo deprotonation and serve as multivalent chelating sites to coordinate with different metal ions. The representative types of MPN-based materials include coatings, films, capsules, nanoparticles, hydrogels, and crystals, which can be prepared by appropriate methods and show specific characteristics and different application prospects [20]. For example, polyphenols can be cross-linked with other substances (e.g., metal ions and boronic acid derivatives) to form hydrogels through the formation of covalent or non-covalent bonds, which can be used to load drugs for tissue repair, drug delivery, and wound healing [43,44]. Dendritic metal–polyphenol coordination crystals can be used as nanoquenchers for the fluorescence sensing of nucleic acids via a quenching mechanism [45]. This section provides a summary of typical preparation methods for MPNs.

Direct self-assembly is the simplest method for the preparation of MPNs. Some metal ions and polyphenols can spontaneously self-assemble into MPNs within seconds or minutes by mixing them under neutral pH conditions. The size of MPNs can be modulated by adjusting the ratio of polyphenols to metal ions. However, rapid coordination kinetics will hinder the formation of well-defined nanoparticles, which is unfavorable in sensing applications. Therefore, different seed agents such as drugs, biomolecules, and polymers have been utilized to accelerate the complexation process and promote the formation of nanostructured MPNs [46]. For instance, poly (ethylene glycol) (PEG) was used as the seeding agent for the preparation of bioactive MPN nanoparticles. During the assembly process, PEG, acting as a seeding agent, can increase the local concentration of metal ions and polyphenols, accelerating the generation of metal–polyphenol complexes. Then, PEG serves as the shielding reagent and the complexes gradually form MPN nanoparticles with well-defined sizes [47]. The chain length or molecular weight (from 1 to 35 kDa) of PEG also affected the growth of MPN nanoparticles. An increased molecular weight of PEG led to the reduction in the size of MPN nanoparticles, which may be attributed to the increased degree of crosslinking and shielding. The smaller MPN nanoparticles exhibit a larger surface area and more reactive metal sites, which is favorable in sensing fields. Nonetheless, the introduction of seeding agents may affect the performance of synthesized MPNs. In 2003, Caruso’s team reported that the type of buffer, such as phosphate buffer, can slow down the coordination kinetics for directly producing well-defined MPN nanoparticles without the use of a template or seeding agent [48]. Meanwhile, they found that the size and morphology of nanoparticles can be controlled by adjusting self-assembly conditions, such as the reaction time, concentration of building blocks, metal-to-ligand ratio, and coordination mode at different pH values.

Polyphenols with intrinsic adhesion properties can adsorb onto various substrates for further complexing with metal ions to form films. Therefore, MPN-coating nanomaterials can be easily prepared through hard template-assisted self-assembly [49]. Generally, this strategy involves two steps: growth of MPNs on the sacrificial template surfaces such as lignin, polystyrene, and CaCO_3_ particles, and then selective removal of the template with the help of a certain solvent or reagent. The morphology and size of the sacrificial template can precisely adjust the geometric shape and average size of the formed MPNs. For example, Ping et al. prepared pH-responsive MPN capsules for anti-cancer drug delivery by using CaCO_3_ as the template [50]. Poly (styrene sulfonate) (PSS)-doped CaCO_3_ particles were used to adsorb a large amount of doxorubicin hydrochloride drugs. Then, the stable MPN shells were deposited on CaCO_3_ particles via the coordination of metal ions and TA molecules in a pH 8.0 buffer solution. After the removal of the CaCO_3_ template using a Tris-acetate buffer or ethylene diamine tetraacetic acid (EDTA), doxorubicin-loaded capsules were successfully synthesized for intracellular delivery.

Emulsion-based interfacial self-assembly is a widely used fabrication method for MPN films and capsules. The size- and rigidity-controllable emulsion template is formed at the interface between two insoluble liquids, such as water and oil. Once the metal ions and polyphenols are mixed in the emulsion, MPNs can spontaneously assemble into films or capsules at the interface or within the emulsion.

The coating layer can change the surface functionality and substrate property by carefully selecting coating materials [51]. Benefiting from the strong adherent ability of phenolic groups, MPNs have been considered multifunctional modifiers for various substrates with hydrophilic or hydrophobic interfaces, such as graphene oxide nanosheets, magnetic nanoparticles, gold nanoparticles (AuNPs), SiO_2_ particles, polyamide membranes, cells, bacteria, viruses, etc. [42,52,53,54,55,56]. Generally, the coating process can be divided into a one-step method or multi-step method. For instance, Andrikopoulos et al. synthesized Zn-EGCG MPN-coated AuNPs via the one-step method by adding Zn^2+^ and EGCG into the AuNPs solution [57]. The as-formed Zn-EGCG MPN-coated AuNPs could modulate amyloid aggregation and reduce its toxicity. The multi-step method involves sequentially incubating substrates with excess polyphenols and metal ions, respectively, ultimately producing thickness-controllable coating films [58].

Inorganic–organic hybrid materials such as crystalline MOFs have been widely used in various fields because of their excellent advantages, which have been comprehensively commented on in many previous reviews [14,15,16]. Compared to crystalline MOFs, MPNs exhibit several inherent advantages for sensing applications. First, the synthesis conditions of MPNs are always mild, simple, and facile. The self-assembly of metal ions and polyphenols can be achieved within minutes in an aqueous solution at room temperature, avoiding the use of hydrothermal conditions and organic solvents. The mild synthesis conditions are beneficial for maintaining the biological activities of enzymes and antibodies, thus facilitating the development of biosensors and wearable devices [59]. Second, the functionalization of MPNs can be engineered by rationally selecting specific metal ions and polyphenols [60]. For example, the intrinsic biological properties (e.g., anti-bacterial and anti-cancer effects) of polyphenols can be retained in MPNs, which are useful for constructing integrated diagnostic and therapeutic platforms. In addition, polyphenols stored in MPNs have a strong reducing ability and can form other nanomaterials in situ on the surface of MPNs, thereby producing hybrid nanocomposites to improve sensing performance. Due to the excellent metal chelating ability of polyphenols, multiple metal ions can be simultaneously integrated into a single type of MPN to regulate the properties of MPNs, such as the catalytic activity, fluorescence property, and redox activity. Third, the surface property of MPNs allows for facile modification with specific chemical and biological species to act as nanoprobes in sensing applications [61]. The numerous phenolic hydroxyl groups on MPNs can facilitate the conjugation of other substances for sensing and biomedical applications through different strategies, such as the catechol–thiol reaction, metal coordination, and boronic acid–catechol complexation. Moreover, the coordinatively unsaturated metal ions on the surface of MPNs can robustly interact with hexahistidine-tagged recombinant proteins and antibodies by metal coordination interactions, while the modified MPNs still preserve their biological activity for sensing and biomedical applications.

## 3. MPNs-Based Sensing Applications

### 3.1. Sensing of Small Molecules and Biological Species

#### 3.1.1. Optical Sensing

**Figure 2:**
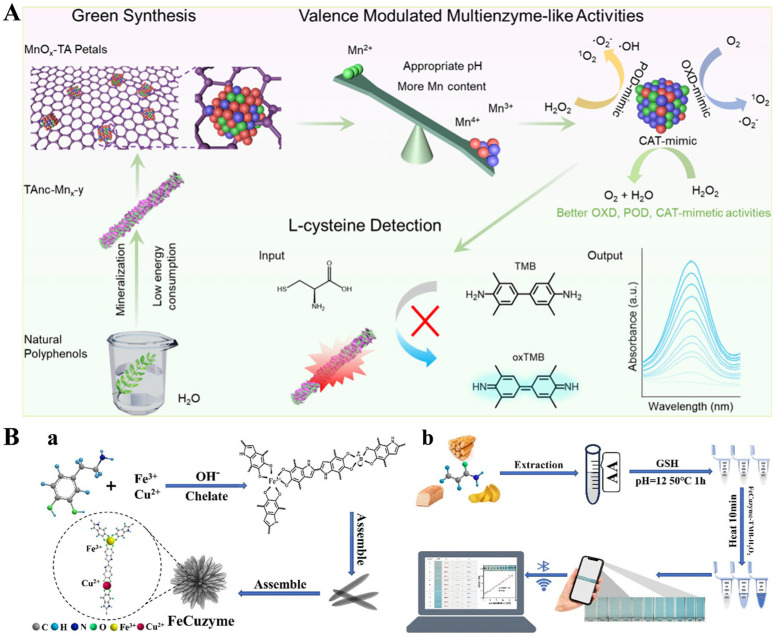
(**A**) Schematic illustration for green preparation of TAnc-Mn*_x_*-*y* and sensing of l-cysteine [68]. Copyright 2024 American Chemical Society. (**B**) Schematic illustration for preparation of FeCuzyme (**a**) and portable detection platform (**b**) [69]. Copyright 2025 Elsevier.

Nanomaterials with enzyme-like properties named nanozymes have attracted considerable attention in bioanalysis due to their excellent catalytic activity, good stability, and low cost [62]. As the most representative example of peroxidase mimetic systems, nanozymes can catalyze the chromogenic reaction between 3,3’,5,5’-tetramethylbenzidine (TMB) and H_2_O_2_ to generate blue-colored oxidized TMB (oxTMB), leading to the solution color change from colorless to blue. Reductive substances such as ascorbic acid, biothiol, and dopamine can hinder the chromogenic reaction, causing a weakened signal for determining these reductive species [63]. Thus, metal ions endow MPNs with different enzyme-like activities for the colorimetric detection of different reductive biomolecules, such as Cu-TA, Co-TA, and Fe-TA nanostructures [64,65,66,67]. In addition, Wei et al. reported a Mn-TA enzyme mimic (TAnc-Mn*_x_*-*y*), with a flower-like shape and multienzyme mimetic properties through the mineralization of MPNs in an aqueous solution (Figure 2A) [68]. The synthesis method is green since water was used as the sole solvent and polyphenol and metal ion were used as the feedstocks. The flower-like structure can enhance the surface area and pore size. The TAnc-Mn*_x_*-*y* mimic displayed excellent oxidase (OXD), peroxidase (POD), and catalase (CAT)-like catalytic activities. It could be used to detect l-cysteine due to its OXD-mimetic activity. Chen et al. prepared a flower-like bimetallic FeCu nanozyme (FeCuzyme) by the metal coordination interaction of Fe^3+^/Cu^2+^ and dopamine (Figure 2B) [69]. The FeCuzyme showed a flow-like shape with 3D catalytic centers and was used for the colorimetric detection of acrylamide based on the TMB/H_2_O_2_ system. The nanozyme catalyzed the oxidation of TMB to yield oxTMB, resulting in the solution color change from colorless to blue. TMB oxidation could be quenched by GSH. However, the quenching could be limited by the thiolene-Michael addition reaction between GSH and acrylamide.

**Figure 3:**
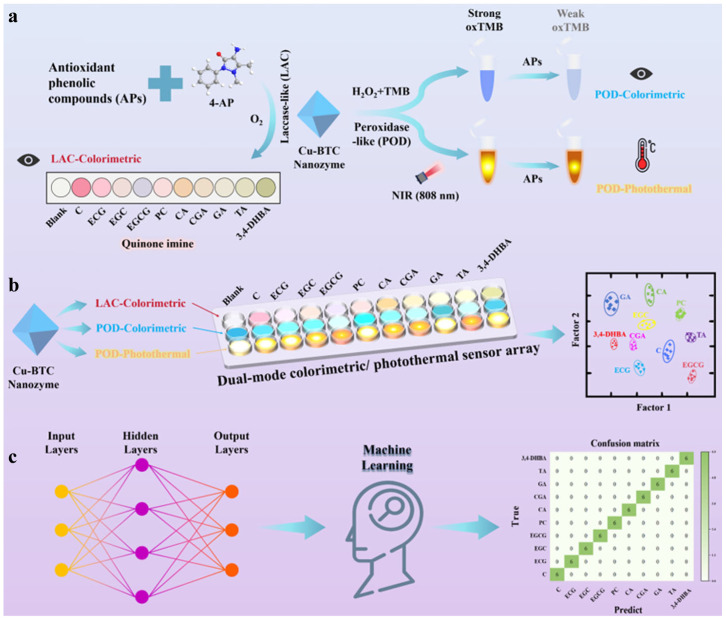
Schematic diagram of (**a**) the mechanism for EPs detection with VA-Cu nanozyme, (**b**) six-channel sensor arrays, and (**c**) machine learning-based nanozyme sensor arrays [71]. Copyright 2024 American Chemical Society.

Detecting endogenous phenolic compounds (EPs) in food is of great significance for evaluating their biological activity and health effects [70]. Jing et al. suggested that vanillic acid-Cu (VA-Cu) nanorods showed peroxidase-like and laccase-like activities and could be used for the discriminant analysis of EPs (Figure 3) [71]. The VA-Cu nanozyme with peroxidase-like behavior catalyzed the oxidation of TMB to yield oxTMB. This reaction was limited by EPs due to their high reducing ability. Meanwhile, the VA-Cu nanozyme with laccase-like behavior could facilitate the oxidation of various EPs, leading to the generation of colored quinone imines. Finally, the VA-Cu nanozyme sensor arrays were combined with artificial neural network algorithms to achieve the identification and prediction of nine EPs in black tea, honey, and grape juice using a smartphone. Simple and real-time methods for monitoring food freshness can upgrade spoilage issues. Recently, Chen et al. we proposed a colorimetric sensor array (CSA) for the intelligent detection of meat freshness based on the multiple competitive coordination of MPNs [72]. The array was fabricated by loading metal–polyphenol solutions on polytetrafluoroethylene (PTFE) membranes. This work achieved the selective and sensitive detection of amines via metal polyphenol–amine interactions. The MPNs formed between various metal ions (Fe^2+^, Fe^3+^, and Cu^2+^) and phenols (protocatechualdehyde, caffeic acid, GA, and TA) exhibited different colors in the presence of total volatile basic nitrogen (TVB-N), allowing for real-time monitoring of the freshness of beef, chicken, fish, and shrimp. Through the combination with the convolutional neural network, an intelligent detection system for meat freshness and an online operating interface was successfully developed. The accuracy for monitoring meat freshness reached at least 99.83%. The innovative colorimetric method has the potential to become a widely applicable and cost-effective tool, helping to address food safety issues and minimize future food waste.

**Figure 4:**
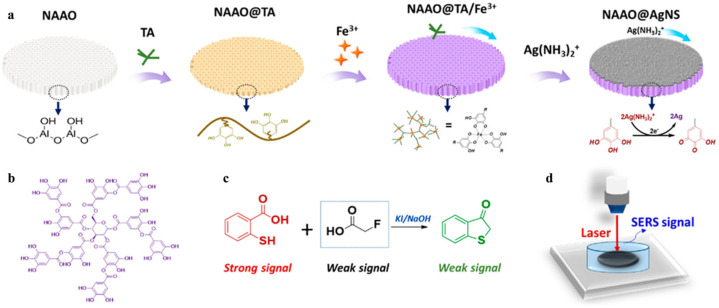
(**a**) Illustration of the formation of TA/Fe^3+^ MPNs on the NAAO via a multistep assembly as well as in situ electroless deposition of AgNS over the NAAO/TA/Fe^3+^. (**b**) Chemical structure of TA. (**c**) Derivative reaction between FAcOH and TSA. (**d**) Proposed SERS detection platform in aqueous environments [76]. Copyright 2021 American Chemical Society.

**Figure 5:**
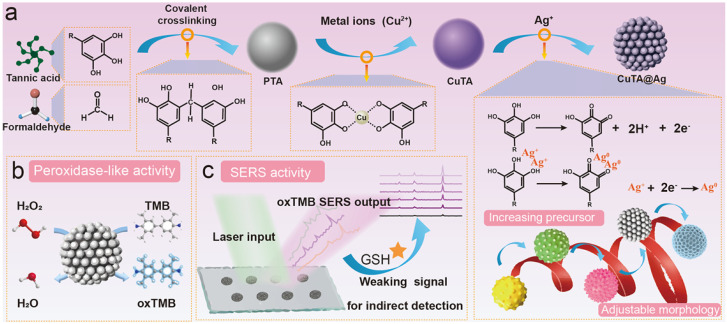
Schematic illustration of (**a**) synthetic process of CuTA@Ag nanostructures, (**b**) CuTA@Ag, and (**c**) SERS detection of GSH in cell samples [77]. Copyright 2023 Elsevier.

Surface-enhanced Raman scattering (SERS) has been popularly used for target detection (e.g., drugs, pesticides, biomarkers, and metabolites) due to its high sensitivity and non-destructive and specific molecular “fingerprint” properties [73]. The fabrication of uniform and reproducible SERS substrates is a vital issue in SERS analysis [74]. MPNs can form at various micro/macro interfaces due to their unique adhesion capability. The reductive polyphenols on MPNs can reduce metal ions to induce the deposition of metal nanostructures on the surface of MPNs. The MPN-supported hybrid nanocomposites can serve as effective SERS substrates to enhance Raman signals [75]. However, it is difficult to sensitively detect substances with low polarization and a Raman cross-section. Chemical derivations or additional reactions provide a promising alternative solution for effective SERS analysis. Sun et al. developed a SERS biosensor for monofluoroacetic acid (FAcOH) detection using Fe^3+^-TA MPNs to coat nanoanodic aluminum oxide film (NAAO) (Figure 4) [76]. The coating layer assisted with the in situ chemical deposition of a highly uniform Ag nanostructure (AgNS) on the NAAO surface. Thiosalicylic acid (TSA) was used as the Raman probe by reacting with FAcOH for target detection. In order to further improve quantitative accuracy, thiocyanate (SCN^−^) was added onto the NAAO@AgNS as an internal standard. The peak intensity ratio of TSA and SCN^−^ (*I*_1035_/*I*_2125_) was intensified with the concentration of FAcOH. Besides the chemical derivation of the target, the nanozyme-catalyzed chromogenic reaction can be combined with SERS detection, especially for reductive targets. For instance, Li et al. synthesized a Ag nanozyme on Cu-TA nanospheres via an interfacial polyphenol reduction method for the SERS determination of GSH [77]. As displayed in Figure 5, Cu-TA nanospheres were facilely prepared via a formaldehyde-assisted metal–ligand crosslinking strategy. Plenty of catechol groups on the surface of nanospheres chelated with silver ions further reduced them for the in situ formation of AgNP-loaded nanospheres (CuTA@Ag). The generated CuTA@Ag exhibited an enhanced peroxidase-like activity compared with CuTA alone. It could catalyze the oxidation of TMB into oxTMB, producing an inherent strong Raman signal. But GSH in samples could reduce oxTMB to Raman-inactive TMB, thereby decreasing the Raman signal. The CuTA@Ag nanostructures have been used to determine GSH in real cell samples with a detection limit of 0.1 μmol/L.

The increasingly serious environmental contamination of plastic has become a global concern. However, the absence of specific groups on plastic makes it difficult to accurately detect plastic in the environment. SERS is expected to become a promising technology to identify and determine plastic in samples [78,79]. Ye et al. reported a SERS sensing platform for monitoring nanoplastic contamination by employing luminescent Zr^4+^-TA MPNs (L-MPNs) to separate and label nanoplastics [80]. Rhodamine B (RhB), serving as a Raman reporter, was enclosed in the MPNs. The labeling of nanoplastics with L-MPNs helped to efficiently separate nanoplastics from liquid media. Nanoplastic at a concentration down to 0.1 μg/mL was determined with a portable Raman instrument. The inherent surface properties and sizes of nanoplastics may affect their specific interactions with L-MPNs (e.g., electrostatic interactions, hydrogen bonding, and van der Waals forces), leading to differential RhB-binding efficiencies. This work found that L-MPN labeling enabled the SERS method with a superior performance in determining a wide array of nanoplastics with different sizes (50–500 nm) and types (e.g., polystyrene, polymethyl methacrylate, and polylactide). In addition, Zeng et al. reported a SERS sensor for the enantioselective identification of chiral molecules based on the chiral surface of the metal–polyphenol framework. The enantioselective identification chiral nanocomposites were fabricated with L-tartaric acid (L-TA), Cu^2+^, and aminothiophenol-anchored AgNP as the chiral recognition selector, signal corrector, and artificial traction skeleton, respectively. With L-/D-cysteine as an example, the chiral recognition selector Cu-L-TA framework showed a stereoselective target recognition ability, which was monitored by SERS technique.

**Figure 6:**
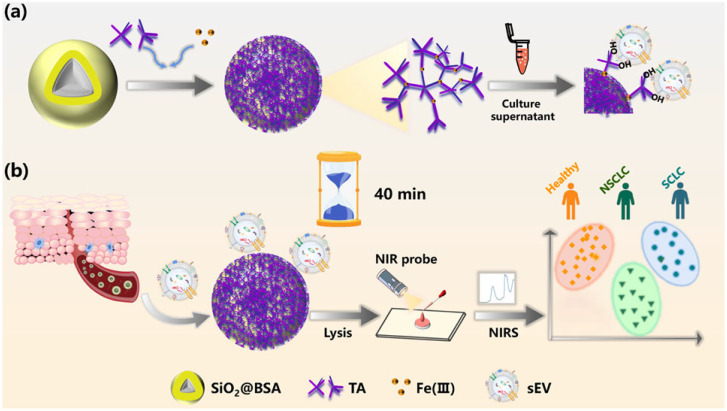
Schematic illustration of fabrication and application of SiO_2_@BSA@Fe-TA_6_ in sEV isolation (**a**,**b**) further downstream analysis article [81]. Copyright 2022 American Chemical Society.

Small extracellular vesicles (sEVs) are increasingly recognized as circulating biomarkers and predictive factors for disease diagnosis. Wang et al. reported a simple technique for the isolation and detection of sEVs based on metal–polyphenol three-dimensional networks [81]. As a proof-of-concept, TA and Fe(III) were used as the polyphenol ligand and metal source (Figure 6). A mesoporous SiO_2_ bead was coated by the TA-Fe(III) network, with bovine serum albumin (BSA) as the additional blocker. The SiO_2_@BSA@Fe-TA_6_ was prepared by a coordination-driven, layer-by-layer self-assembly method. It could be used for the universal capture of sEVs in distinct cellular and plasmatic samples. The capture efficiency (~85.4%) is comparable to that of immunoreaction technology and higher than that of the ultracentrifugation method. Finally, this strategy was used for the clinical screening of different subtypes of lung cancer patients by combining near-infrared spectroscopy (NIRS) with chemometrics.

#### 3.1.2. Electrochemical Sensing

**Figure 7:**
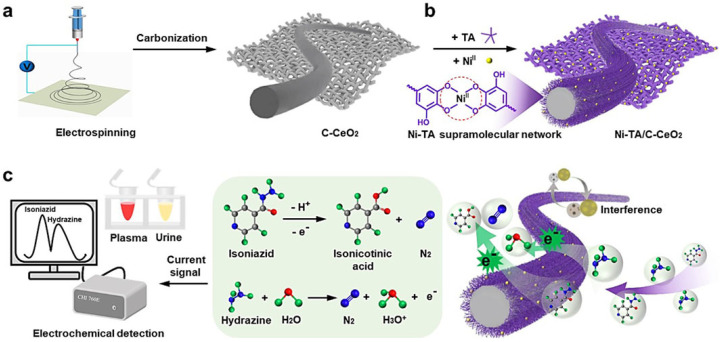
Schematic illustration of (**a**) synthetic process of electrospun-derived C-CeO_2_ nanofiber; (**b**) the formation of Ni-TA MPN on C-CeO_2_ nanofiber; and (**c**) the possible mechanism of Ni-TA MPN-coated C-CeO_2_ nanofibers for electrochemical detection of isoniazid and hydrazine [83]. Copyright 2024 Elsevier.

Electrochemical sensors for the detection of small molecules have several unique advantages, including low cost, fast response, ease of operation. Their detection sensitivity mainly relies on the mass transport of electroactive substances on the electrode surface. MPNs have been used to modify sensing electrodes and improve the mass transport of electroactive species [82]. For example, Feng’s group reported several electrochemical sensors for the detection of isoniazid, hydrazine, and glucose based on Ni-TA MPN-coated nanomaterials [82,83]. As illustrated in Figure 7, the electrospun-derived C-CeO_2_ nanofibers were coated with Ni-TA MPNs for the detection of isoniazid and hydrazine. The electronegative and hydrophilic Ni-TA MPNs could accelerate the mass and electron transfer in the electrooxidation of both isoniazid and hydrazine, thereby enhancing the electrochemical signal. The method has been used to determine isoniazid and hydrazine in human-collected plasma and urine samples, with a detection limit of 12 nM and 8 nM, respectively. In their other work, Ni-TA-coated C-Cr_2_O_3_ nanoparticles were prepared and used as electrode modifiers to determine glucose and hydrazine by accelerating the charge transfer and enhancing the amperometric response [82]. The detection limit of this sensor was 0.02 μM for glucose and 0.04 μM for hydrazine.

**Figure 8:**
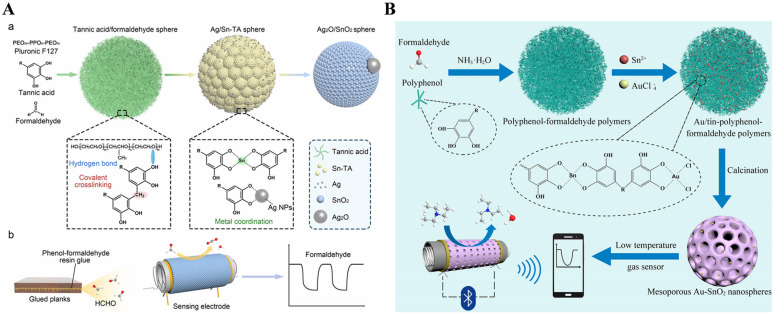
(**A**) Schematic illustration of (**a**) synthesis process of Ag_2_O/SnO_2_ nanospheres and (**b**) actual sample detection [84]. Copyright 2022 American Chemical Society. (**B**) Schematic illustration for the self-template synthesis of mesoporous Au-SnO_2_ nanospheres and the low-temperature detection of TEA [86]. Copyright 2022 Elsevier.

Self-template strategies for the preparation of mesoporous metal oxide nanomaterials by direct thermal decomposition of metal–organic coordination polymers have attracted widespread attention. Wei’s group have synthesized spherical mesoporous SnO_2_, Ag_2_O/SnO_2_, and Au-SnO_2_ nanospheres from Sn-polyphenol-formaldehyde polymers for the sensing of gaseous ethanol, formaldehyde, and triethylamine [84,85,86]. The Sn-polyphenol-formaldehyde polymers were synthesized by a sol–gel process using TA, formaldehyde, and Sn^2+^ ion as the ligand crosslinking agent, and metal source, respectively. The block copolymers could regulate the polymerization process and promote the formation of uniform spheres with a diameter of ~200 nm. Mesoporous Ag_2_O/SnO_2_ nanospheres were synthesized using TA as the polyphenol ligand and reducing agent, followed by further thermal decomposition of the frameworks at a high temperature (Figure 8A) [84]. The spherical morphology was well preserved after the modification of silver nanoparticles, indicating that the presence of Ag^+^ ions did not affect the formation of Sn-TA spheres. The decoration of Ag_2_O could enhance the adsorption energy toward formaldehyde, facilitating the sensing of formaldehyde with a detection limit of 23.6 ppb at a lower working temperature. No significant change in the resistance was observed even after eight cycles, indicating good reproducibility of the Ag_2_O/SnO_2_ sensor. In addition, mesoporous Au-SnO_2_ nanospheres were prepared using TA as the chelating agent as well as Sn^2+^ and HAuCl_4_ as the metal sources (Figure 8B) [86]. The Au/Sn-polyphenol-formaldehyde spheres were used as the precursors to prepare mesoporous Au-SnO_2_ crystalline frameworks by direct calcination. The addition of Au species did not induce an obvious change in the morphology of mesoporous spheres. The nanospheres could be used for the sensing of triethylamine, with a detection limit of 0.11 ppm based on the adsorption or reaction-induced resistance change.

Electrochemiluminescence (ECL) involves the generation of luminescence through the sequential completion of an electrochemical reaction and chemiluminescence reaction at/near the electrode surface [87]. Zou et al. developed an ECL biosensor for monitoring histone acetyltransferase activity and the screening of inhibitors by using Fe^3+^-TA complex-coated AuNPs as nanoprobes (AuNPs@TA-Fe) [88]. The acetylation of the substrate peptide by histone acetyltransferase limited the hydrolysis of peptides by trypsin, allowing for the attachment of nanoprobes on the electrode surface by hydrophobic and hydrogen-bonding interactions. This decreased the ECL signal in the luminol solution since the superoxide dismutase-mimetic TA eliminated the production of ROS. Based on the change in ECL intensity, the method was further applied for detecting histone acetyltransferase activity in cell lysates and the screening of potential inhibition drugs.

### 3.2. Immunoassays

#### 3.2.1. Optical Immunoassays

**Figure 9:**
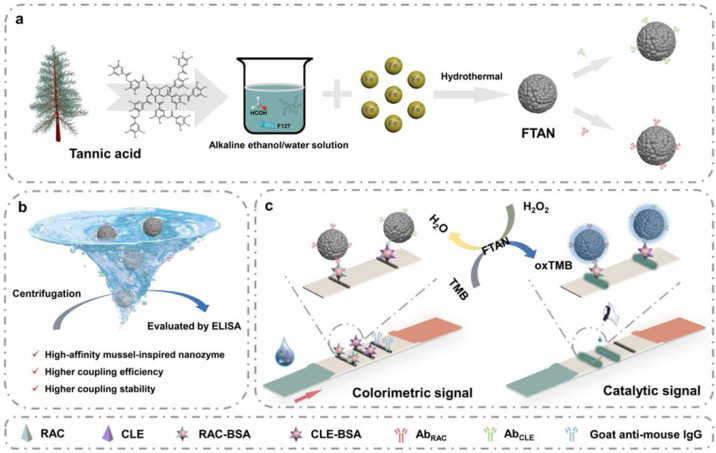
(**a**) Schematic illustration of synthesis and fabrication of bioresource-derived PTAN-based immuno-network. (**b**) Comparison of monoclonal antibody required for indirect probe-based immune-network versus traditional direct label pattern [93]. Copyright 2022 Elsevier.

Immunoassays have become powerful analytical tools for the sensitive determination of trace targets based on the highly specific recognition between antigens and antibodies [89]. According to the types of output signals, immunoassays can be divided into colorimetric, fluorescent, electrochemical, ECL, and photoelectrochemical methods. Thereinto, colorimetric immunoassays have attracted considerable attention due to their high simplicity and efficiency [90]. The color change in the solution can be qualitatively or semi-quantitively observed with the naked eye and then quantitively measured by spectroscopy. In order to improve the sensitivity of colorimetric immunoassays, enzymes, plasmonic noble metal nanomaterials, and nanozymes have been used as nanolabels or chromogenic substrates for signal amplification [91]. Wang’s group reported several colorimetric immunoassays based on the signal amplification of AuNPs and peroxidase-like MPNs [92,93,94]. For example, they reported a lateral flow immunoassay (LFIA) tool for ractopamine (RAC) and clenbuterol (CLE) detection using the Fe-TA nanozyme (FTAN) for colorimetric/catalytic dual readouts and dual-semiquantitative detection (Figure 9) [93]. The nanozyme exhibited excellent coupling efficiency and stability for the immobilization of antibodies in view of its mussel-inspired adsorption ability. Meanwhile, they designed a peroxidase-like Fe-GA nanozyme (FGN) for colorimetric immunochromatographic analysis [94]. The nanozyme served as a recognizer for antibody immobilization and a generator for signal readout and amplification. The on-site detection was achieved with the assistance of smartphone and principal component analysis. As a proof-of-concept, clenbuterol was determined in the range of 0–6 ng/mL, with a detection limit of 0.172 ng/mL.

**Figure 10:**
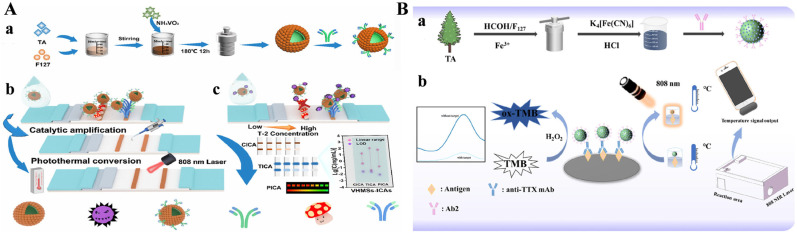
(**A**) Schematic illustration of (**a**) synthetic process of VHMSs, (**b**) principle for *T*-2 detection, and (**c**) results of three modes [98]. Copyright 2024 American Chemical Society. (**B**) Schematic illustration of (**a**) synthetic procedure of antibody-modified FTAN@PB and (**b**) working principle of colorimetric and photothermal dual-mode immunoassays of tetrodotoxin [99]. Copyright 2025 Elsevier.

In recent years, colorimetric methods have been successfully combined with photothermal techniques to develop multi-mode immunoassays [95,96]. Such immunoassays show a higher sensitivity and accuracy than traditional single-mode methods. A lot of nanolabels of multi-mode immunoassays have been rationally designed with an enzyme-like catalytic performance and photothermal conversion ability, such as Prussian blue (PB), anisotropic gold nanostructures, graphene oxide, metal sulfide nanomaterials, etc. [97]. Vanadium can not only coordinate with TA to form MPNs but also serve as a nanozyme and a photothermal agent. Wu et al. developed a triple-readout immunochromatographic assay method for *T*-2 detection using hollow vanadium nanomicrospheres (VHMSs) as three-in-one multifunctional labels (Figure 10A) [98]. In this study, VHMSs were prepared via a formaldehyde-assisted metal–ligand crosslinking strategy based on the coordination of the vanadium ion and TA. They showed a darker original color and acted as ideal signal tracers. To further widen the detection range, VHMSs were used as peroxidase-like nanozymes to catalyze the chromogenic reaction between TMB and H_2_O_2_, deepening the color for signal amplification. More importantly, VHMSs with an excellent photothermal effect could cause the concentration-dependent temperature change under 808 nm laser irradiation, realizing the photothermal detection mode with high sensitivity. This immunoassay in the photothermal mode can detect *T*-2 with a detection limit of 2 pg/mL. In addition, MPNs can be combined with other nanomaterials that possess catalytic and photothermal properties for the development of multimode immunoassays. For example, Raza et al. reported a PB-anchored Fe(III)-TA composite-based colorimetric and photothermal immunoassay platform for tetrodotoxin detection (Figure 10B) [99]. In this work, Fe(III)-TA coordination particle (FTAN) was synthesized and served as the support for the in situ growth of PB on the particle surface. The FTAN@PB possessed peroxidase-like catalytic activity and photothermal properties. After modification with goat anti-mouse IgG, the nanolabel was introduced into a competitive immunoassay for dual-mode detection. The FTAN@PB-catalyzed oxidation of TMB by H_2_O_2_ provided a colorimetric signal. FTAN@PB and oxTMB led to the temperature increase due to their photothermal effect under 808 nm laser irradiation. The temperature change was recorded by a smartphone-based infrared camera. This dual-mode immunoassay for tetrodotoxin detection achieved detection limits of 0.26 and 0.44 ng/mL with the colorimetric and photothermal modes, respectively.

**Figure 11:**
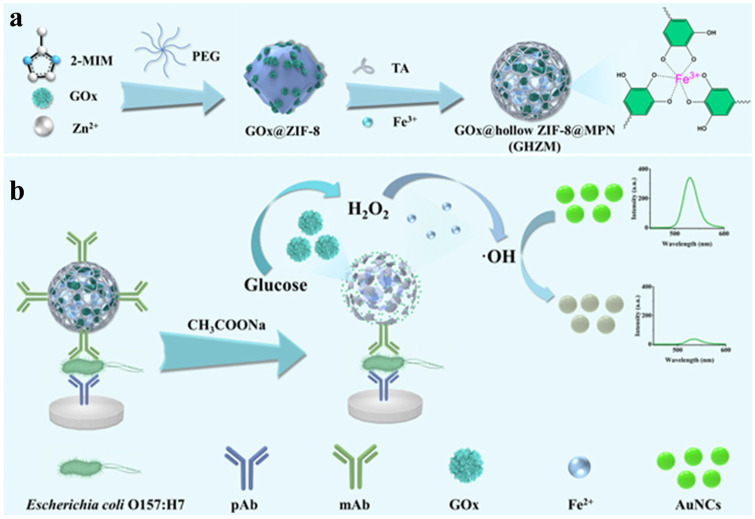
Schematic illustration of the synthetic procedure of GHZM (**a**) and the detection process of GHZM-FELISA for *E. coli* O157:H7 (**b**) [101]. Copyright 2024 Elsevier.

Fluorescent immunoassays have been the most popularly used analytical methods due to their characteristics of fast response, high sensitivity, and excellent stability [100]. Various fluorescent materials have been employed as signal reporters for immunoassays, including organic dyes, noble metal nanoclusters, carbon dots, and polymer dots. Jing et al. developed a fluorescence immunoassay method for the detection of *Escherichia coli* O157:H7 using MPN-coated hollow ZIFs@GOx as a “three-in-one” signal label (Figure 11) [101]. In this study, ZIF-8 was used in situ to encapsulate GOx in the presence of polyethylene glycol. The addition of TA produced free protons that can etch ZIF-8 into hollow nanostructures, leading to the retention of the conformational freedom and catalytic activity of GOx. Then, TA adsorbed on the surface of GOx@ZIF-8 was coordinated with Fe^3+^ to form a network shell, which could prevent the degradation of GOx@ZIF-8 and facilitate the immobilization of antibodies. In the presence of glucose and O_2_, GOx catalyzed the generation of H_2_O_2_, and the acid condition from the enzymatic product gluconic acid promoted the disassembly of MPNs. The released Fe^3+^ was reduced into Fe^2+^ by TA, further catalyzing the decomposition of the self-produced H_2_O_2_ into ⋅OH via the Fenton reaction. Gold nanoclusters (AuNCs) with a green emission were used as fluorescent indicators, which can be oxidized by ⋅OH to induce fluorescence quenching. This fluorescence immunoassay can sensitively detect *Escherichia coli* O157:H7 with a detection limit of 1.35 × 10^3^ CFU/mL. However, most of them faced the problem of fluorescence quenching at high concentrations or solid states.

**Figure 12:**
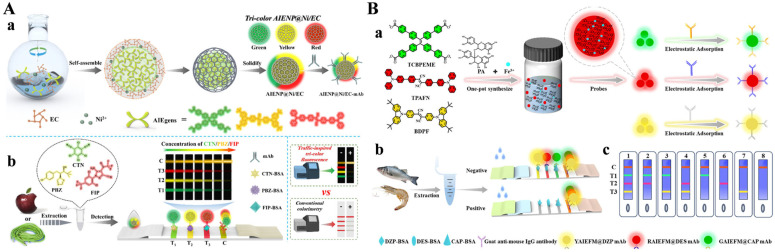
(**A**) Schematic illustration of (**a**) synthesis of three-color AIENP@Ni/EC and (**b**) simultaneous quantitative detection of CTN, PBZ, and FIP in apples and cowpeas based on T-FLFIA, compared to conventional colorimetric M LFIA [106]. Copyright 2024 Elsevier. (**B**) Schematic illustration of (**a**) one-pot synthesis of AIEFMs and preparation of AIEFM@mAb probes, (**b**) simultaneous detection of CAP, DES, and DZP, and (**c**) qualitative test results [107]. Copyright 2025 Elsevier.

Unlike traditional fluorophores, aggregation-induced emission (AIE) luminogens (AIEgens) can produce high fluorescence intensity even at high concentrations or in an aggregated state [102]. AIE nanostructures can be integrated with MPNs to serve as signal tracers for fluorescence bioimaging and immunoassays [103,104]. For example, Chen et al. reported an LFIA method by using biocompatible Zr-TA MPN to modify AIE fluorescence microspheres (AIEFM) for the immobilization of antibodies [105]. Carbendazim was determined by the AIEFM@MPN-LFIA with a detection limit of 0.019 ng/mL. Deng’s group reported a traffic signal-inspired fluorescent lateral flow immunoassay (LFIA) platform by integrating AIENPs and MPNs (Figure 12A) [106]. The AIENP was coated with the Ni^2+^-epicatechin (Ni/EC) network by a self-assembly method and served as a fluorescence probe. The AIENP@Ni/EC with three distinct colors and fluorescence signals (green, yellow, and red) could be prepared by coating various AIEgens. By direct conjugation of AIENP@Ni/EC with the target-specific antibody via electrostatic interactions, the multicolor fluorescence LFIA was used for the simultaneous detection of chlorothalonil (CTN), paclobutrazol (PBZ), and fipronil (FIP) in cowpea and apple samples. In addition, Deng’s group reported a multiplex immunochromatographic assay platform for the simultaneous detection of chloramphenicol (CAP), diethylstilbestrol (DES), and diazepam (DZP) using triple-color AIE fluorescent microspheres as signal labels (Figure 12B) [107]. In this work, AIE fluorescent microspheres were prepared through a facile one-pot method based on the aggregation of three AIEgens with green, red, and yellow emissions and the formation of Fe^3+^-proanthocyanidin networks. Then, the produced AIE fluorescent microspheres were directly modified with antibodies without other coupling procedures.

#### 3.2.2. Electrochemical Immunosensors

**Figure 13:**
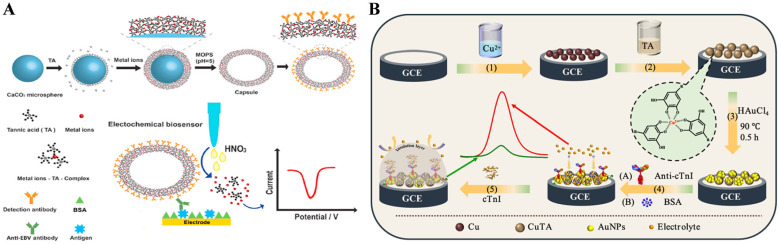
(**A**) Schematic illustration of the working principle of the metal phenolic capsules-based electrochemical immunoassay for EB virus infection [110]. Copyright 2020 Elsevier. (**B**) Schematic illustration for the preparation and detection process of the CuTA@Cu-based cTnI electrochemical immunosensor [111]. Copyright 2024 American Chemical Society.

Among various existing immunoassays, electrochemical immunosensors have attracted widespread attention for the detection of trace analytes due to their high sensitivity, fast response, low cost, and ease of miniaturization [108,109]. Different functional nanomaterials have been utilized to develop high-performance electrochemical immunosensors. MPNs contain a large amount of metal ions that can directly provide electrochemical signals for signal readouts and/or serve as the electrode modifiers for biomolecule immobilization. Huang et al. developed a soft metal–polyphenol capsule-mediated electrochemical immunoassay platform for sensitive detection of the Epstein–Barr (EB) virus infection (Figure 13) [110]. The metal–polyphenol capsule was synthesized using the micro-sized CaCO_3_ microsphere as the hard template, followed by the dissolution of the template in an acid condition. Then, the antibody-modified metal–polyphenol capsule participated in the formation of the sandwich immunocomplex on the electrode surface. The metal–polyphenol capsule with a micrometer size released a tremendous number of Pb^2+^ ions, which could be quantitively determined via anodic stripping voltammetry (ASV). MPNs contain a large number of coordinatively unsaturated metal ions on their surface, facilitating the facile immobilization of antibodies on their surface. Liu et al. developed an electrochemical immunosensor for the detection of cardiac troponin I (cTnI) using copper (II)-tannic acid@Cu (CuTA@Cu)-modified glassy carbon electrodes (Figure 13B) [111]. The redox-active nanocomposite of CuTA@Cu was prepared by the electrodeposition of metallic copper on the electrode surface. Then, HAuCl_4_ was reduced in situ into AuNPs by the reducible catechol group in TA. The AuNPs/CuTA@Cu composites served as bifunctional matrixes for antibody immobilization and signal output. The immunoreaction between cTnI and its antibody caused a significant decrease in the electrochemical signal of CuTA@Cu. The immunosensor could be used to determine cTnI in human serum samples with a detection limit of 0.65 fg/mL.

**Figure 14:**
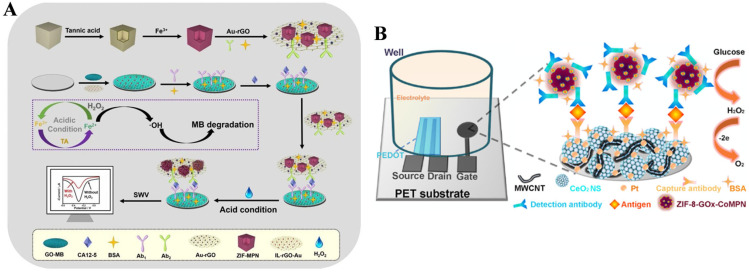
(**A**) Schematic illustration of an electrochemical immunoassay for CA12-5 based on the coating of MPNs on ZIF-8 and TA-assisted cyclic conversion of Fe(III)/Fe(II) [112]. Copyright 2020 Elsevier. (**B**) Schematic illustration of OECT immunosensor for the detection of VEGF_165_ by ZIF-8-GOx-CoMPN nanoprobe as the signal label [113]. Copyright 2023 Elsevier.

In addition, MPNs with enzyme-like catalytic activities can also be used as electrocatalytic labels in electrochemical immunoassays for signal amplification. Zhang et al. reported the electrochemical immunoassay of carbohydrate antigen 12-5 (CA12-5) based on the coating of MPNs on ZIF-8 and the TA-assisted cyclic conversion of Fe(III)/Fe(II) (Figure 14A) [112]. As shown in Figure 13A, ZIF-8 was used as the template to form ZIF-MPN and further adsorbed on the surface of the Au-rGO composite (Au-rGO/ZIF-MPN). In the presence of CA12-5, the antibody-modified Au-rGO/ZIF-MPN formed the immunocomplex on the MB-accumulated electrode surface. After the addition of acidic H_2_O_2_, the MPN released plenty of Fe(III) ions that could be further reduced into Fe(II) by TA. Fe(II) could catalyze the decomposition of H_2_O_2_ into ⋅OH, and Fe(II) was oxidized into Fe(III). Under the cyclic conversion of Fe(III)/Fe(II), the produced ⋅OH could cause the degradation of MB and the decline of the electrochemical signal. The electrochemical immunoassay exhibited a linear detection range from 0.05 mU/mL to 500 U/mL for CA12-5 with a detection limit of 0.0023 mU/mL. In addition to serving as electroactive or electrocatalytic labels, MPNs and their coating materials can also be used as carriers to load natural enzymes for signal amplification. Tang’s group developed an organic electrochemical transistor (OECT) immunosensor for the detection of vascular endothelial growth factor 165 (VEGF_165_) by using zeolitic imidazolate framework-glucose oxidase-CoMPN (ZIF-8-GOx-CoMPN) nanoprobes as the signal label (Figure 14B) [113]. The porous CoMPN on the ZIF-8-GOx surface prevented enzyme activity loss and served as the support for antibody immobilization. The produced H_2_O_2_ by the enzyme–catalytic reaction was oxidized at the platinum-loaded CeO_2_ nanosphere–multiwall carbon nanotube (Pt-CeO_2_ NS-MWCNT)-modified working electrode. This OECT immunosensor showed high sensitivity due to the excellent performance of the nanoprobe for enzyme loading and the OECT device for H_2_O_2_ detection.

**Figure 15:**
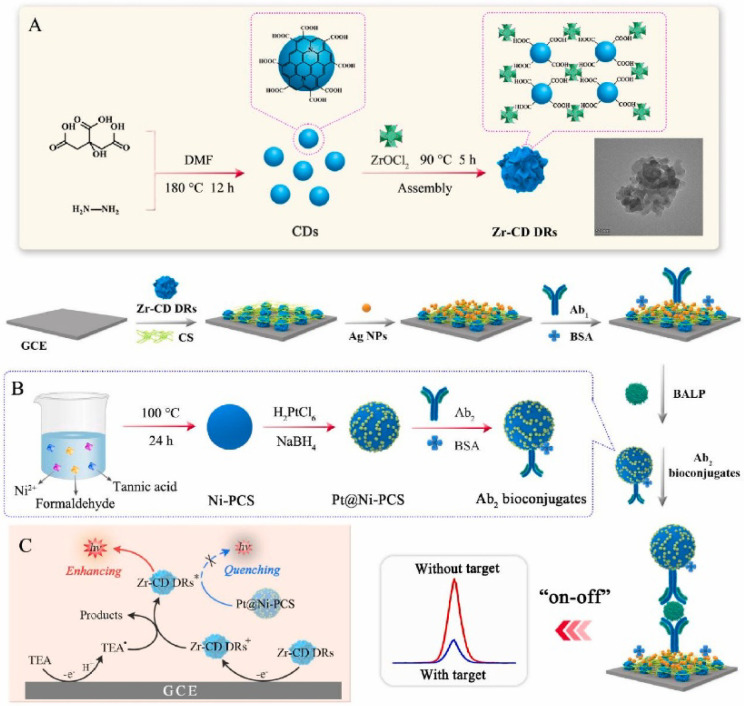
Schematic illustration of an “on-off” ECL immunosensor for sensitive detection of bone alkaline phosphatase using Zr-CD DR as signal label and Pt@Ni-PCS as quencher [115]. Copyright 2023 Elsevier. (**A**) The preparation of Zr-CD DRs, (**B**) the preparation of Pt@Ni-PCS-Ab_2_ bioconjugates, and (**C**) the possible ECL reaction mechanism.

ECL immunoassays show a low background signal, wide linear range, and excellent anti-interference ability by combining the advantages of electrochemical and luminescent immunoassays [114]. Lin et al. developed an ECL immunosensor for bone alkaline phosphatase (BALP) detection using carbon dot dendrimer (CD DR) as the signal label and Pt nanoparticle-functionalized Ni-phenolic coordination sphere (Pt@Ni-PCS) as the quenching label (Figure 15) [115]. In this work, the CD DR was prepared through the coordination-induced self-assembly of carboxyl group-rich N-doped CD and zirconium oxygen clusters. The Ni-PCS was prepared by a formaldehyde-assisted metal–ligand crosslinking reaction. Due to the efficient quenching effect of Pt@Ni-PCS toward the Zr-CD DR/triethyl amine-based ECL system, the immunosensor showed a low detection limit (24.9 fg/mL).

### 3.3. Bioimaging

**Figure 16:**
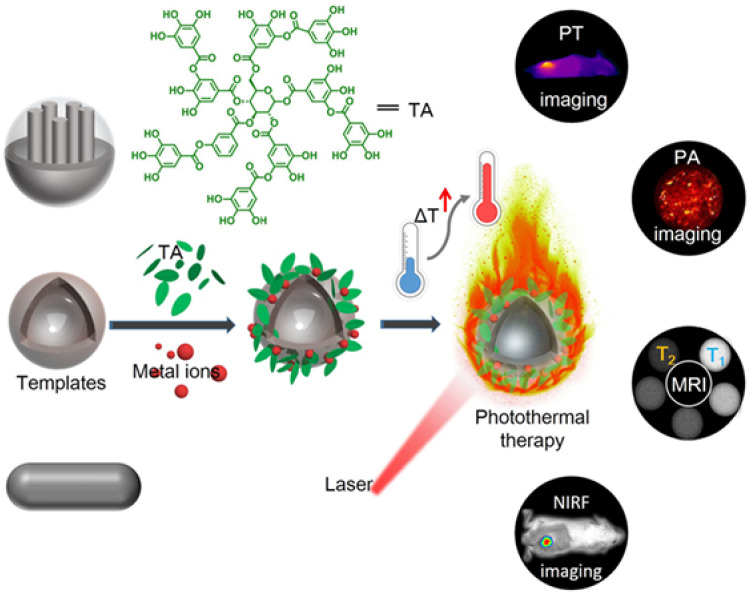
Schematic illustration of the cooperation of adhesive MITAs with diverse templates for advanced applications [120]. Copyright 2018 American Chemical Society.

Bioimaging technology is a key tool for biological analysis, which can enable researchers to understand the functions and structures of organs, tissues, and cells at different depths [116]. MPNs with specific metal ions (e.g., Fe^3+^, Gd^3+^, and Cu^2+^) can be employed for bioimaging applications, such as positron emission tomography, magnetic resonance (MR), and photoacoustic imaging (PAI) [117,118]. In addition, the photothermal effect of MPNs can further provide the possibility of photothermal imaging (PTI) and imaging-guided phototherapy [119]. Liu et al. prepared a series of metal ion/tannic acid-assembled photothermal materials (MITAs) with Fe^3+^, V^3+^, and Ru^3+^ as the metal sources (Figure 16) [120]. Compared with other photothermal agents, MITAs have the advantages of green synthesis, facile incorporation of diagnostic metal ions, and topology-independent adhesion. A variety of nanoengineering can be easily obtained through the self-adhesion of MITAs on different templates due to their adhesive property. This enables MITAs to be highly suitable as photothermal platforms for versatile combination with other therapeutic methods and imaging techniques. Poly (lactic-co-glycolic acid)-based polymeric nanospheres and nanovesicles and mesoporous silica nanoparticles were used as the templates to prepare Fe-TA coated MITAs. The effects of MITAs for photo-responsive applications such as tumor-specific photothermal (PT) and photoacoustic (PA) imaging were investigated. To further prove the potential of MITAs as versatile platforms, PNV-supported Fe-TA (PNV@Fe-TA) networks were prepared and used for T_1_ and T_2_-weighted dual-modal magnetic resonance imaging (MRI) by additionally doping Mn^2+^, as well as in vivo near-infrared fluorescence (NIRF) imaging by encapsulating hydrophilic fluoroprobes.

**Figure 17:**
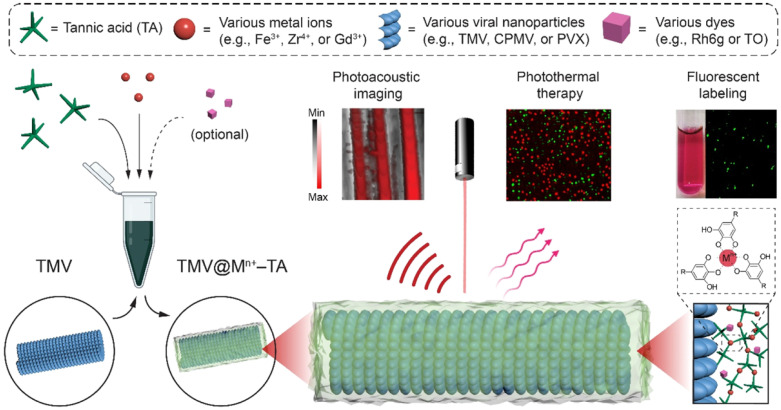
Schematic illustration of synthesis of MPN coating on plant VNPs and biomedical applications [122]. Copyright 2022 American Chemical Society.

Organic dyes can be integrated into MPNs for fluorescence bioimaging [121]. Plant viral nanoparticles (VNPs) are a type of promising biogenetic nanosystem for the delivery of therapeutic, immunotherapeutic, and diagnostic agents. Wu et al. reported a versatile strategy for functionalization of plant VNPs by coating MPNs and labeling organic dyes (Figure 17) [122]. This can enable plant viruses (e.g., tobacco mosaic virus (TMV), cowpea mosaic virus, and potato virus X) with additional functions such as photothermal transduction, photoacoustic imaging, and fluorescent labeling to dope different components in MPNs. As a typical example to prove the potential of this strategy, TMV was chosen as a viral substrate. Coating TMV with Zr^4+^-TA and rhodamine 6G resulted in a strong fluorescence peak at 555 nm. The photothermal conversion efficiency increased from 0.8% to 33.2%, and the photoacoustic performance was improved with a detection limit of 17.7 μg/mL when TMV was coated with Fe^3+^-TA. Furthermore, the TMV@Fe^3+^-A nanohybrids exhibited an excellent cytocompatibility and cell-killing performance under 808 nm irradiation. This work suggested that the MPNs-coated plant VNPs with multifunctionality and biocompatibility show promising therapeutic applications.

### 3.4. Wearable Devices

**Figure 18:**
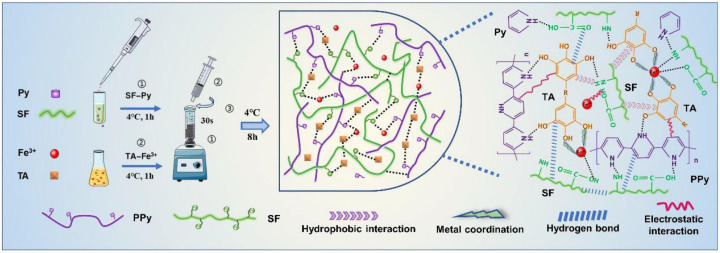
Schematic diagram of the preparation process and gelation mechanism of the SF/TA@PPy conductive hydrogel [127]. Copyright 2022 Elsevier.

Flexible, wearable sensors based on conductive hydrogels have been extensively utilized in health monitoring and human motion because of their high conductivity and cost-effectiveness [123,124,125]. MPNs can be introduced into the formation of hydrogels to promote polymer crosslinking and endow hydrogels with self-repair and wet adhesion capabilities [126]. For this consideration, Zheng et al. prepared a polypyrrole (PPy)-based conductive polymer by introducing silk fibroin (SF) and TA in the gel networks by in situ polymerization (Figure 18) [127]. Mechanical properties of the SF/TA@PPy conductive hydrogel were enhanced due to the multiple dynamic reversible non-covalent bonds (e.g., metal–catechol coordination bond, hydrogen bond, hydrophobic, and electrostatic interactions). In addition, the hydrogel showed good stretchability, skin compliance, and anti-bacterial and biocompatibility properties. It is suitable to be used as an adhesion sensing material for the design of wearable strain devices.

**Figure 19:**
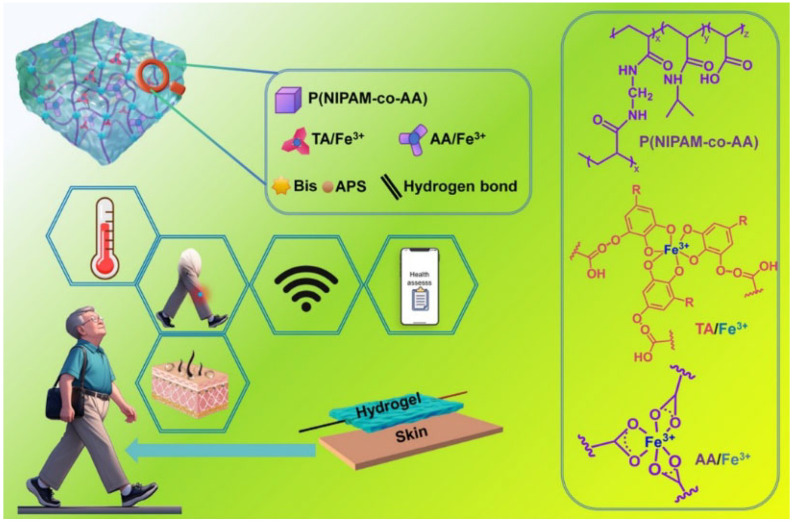
Design and application of multi-signal responsive PNATF ion-conducting hydrogel as flexible wearable sensor [130]. Copyright 2022 American Chemical Society.

In addition to monitoring the change in biomolecules, it is promising to develop wearable sensors for the simultaneous detection of temperature, pH, and motion based on conductive MPN hydrogels [128,129]. For example, Liu et al. prepared a multi-responsive ionic conductive hydrogel with an excellent mechanical property and self-healing ability by the formation of interwoven network structures (Figure 19) [130]. The skeletons were constructed through the co-crosslinking of *N*-isopropylacrylamide (PNIPAM) and acrylic acid (PAA) monomers. The interwoven structures were formed by interpenetrating the Fe-TA networks with basic backbones. The abundant catechol groups in TA molecules endowed the hydrogel with an adhesion strength of 7.06 kPa. No residue was peeled off when the hydrogel was applied to human skin. The ionic conductive hydrogel showed great application potential as a temperature-sensitive sensing material for measuring human movement and environmental temperature and monitoring fever and abnormal thermotherapy.

## 4. Conclusions

The chemical diversity and structural tunability of metal ions and polyphenols have, to some extent, enabled the rational design and controllable self-assembly of MPNs with different functions. As a type of revolutionary inorganic–organic hybrid material with excellent performance, MPNs have many potential applications in the sensing fields, including the direct detection of small molecules and biological species, immunoassays, bioimaging, and wearable devices. This article discussed the typical preparation methods and advantages of MPNs. In addition, the important progress of MPN-based sensing applications was emphasized, which may provide inspiration for the development of novel sensing materials and methods.

Although a variety of MPN-based sensing applications have been implemented, there are still several challenges that need to be addressed. First, multiple phenolic groups in one polyphenol molecule may limit the ordered arrangement of coordination networks, ultimately impacting the formation of well-defined nanomaterials. In addition, only a small fraction of natural polyphenols were used to prepare MPNs for sensing applications. Second, the majority of MPNs are mono-metallic at present. Accordingly, multi-metallic MPNs are expected to show more unique multifunctional properties through synergistic effects, which would be very attractive in sensing fields. Third, the biocompatibility of MPNs is a critical factor for the success of sensing applications. The uncontrolled release of metal ions in MPNs (e.g., Cu^2+^, Fe^3+^, and Co^2+^) may lead to potential toxicity at high concentrations. Finally, in the context of artificial intelligence, the optimal combinations of metal ions and polyphenols should be systematically investigated. The relationship between molecular structure and nanostructure can be analyzed with the aid of graph neural networks, guiding the synthesis of MPNs for improving sensor performances.

In summary, benefiting from the diversity of types and functions of metal ions and polyphenols, MPNs exhibit various excellent properties and have enormous potential in sensing applications. With further development of material science and biochemical analysis technology, we believe that more and more MPN-based materials can be rationally designed and utilized as multifunctional platforms for advanced sensing applications. Through the joint efforts of multiple research fields, such as material science, computer science, and analytical chemistry, significant achievements are expected to predict experimental conditions and understand structure–property relationships. Computational calculations and automated machine learning may be powerful tools for developing MPN-based tailored materials and new methods.

## Data Availability

No new data were created.
